# Shared labor—Public Private Partnerships for Maternal Health Equity

**DOI:** 10.1093/haschl/qxag038

**Published:** 2026-02-12

**Authors:** Rasheca Logendran, Meghana Sai Iragavarapu, Halle Tecco

**Affiliations:** Harvard Medical School, Boston, MA 02115, USA; Harvard Business School, Cambridge, MA 02115, USA; Harvard T.H. Chan School of Public Health, Boston, MA 02115, USA; Duke Medical School, Durham, NC 27710, USA; Harvard Medical School, Boston, MA 02115, USA

**Keywords:** FemTech, maternal health equity, public private partnerships, outcome-indexed reimbursement, Medicaid

## Abstract

The United States faces a worsening maternal health crisis, with mortality rates far exceeding those of peer nations and persistent inequities for Black, Indigenous, and rural women. Medicaid finances over 40% of U.S. births, yet fiscal pressure, heightened scrutiny of program spending, and political resistance to benefit expansion constrain states’ ability to adopt new maternal health interventions. In this environment, outcome-indexed public-private partnerships (PPPs) offer a pragmatic pathway to advance maternal health equity while tying expenditures to measurable value. This paper examines the promise and limitations of emerging FemTech innovations such as virtual doula care, remote patient monitoring, and AI-enabled risk-assessment—and the structural barriers that impede their adoption in Medicaid. We argue that innovation failures reflect misaligned incentives rather than lack of technological potential. To address this gap, we propose a coordinated policy framework that aligns the Advanced Research Projects Agency for Health (ARPA-H), the Centers for Medicare & Medicaid Services (CMS) Transforming Maternal Health Model and Medicaid Section 1115 waivers across the innovation lifecycle. This approach enables disciplined experimentation, independent evaluation, and scalable deployment of equity-focused technologies within Medicaid, safeguarding maternal health gains under constrained fiscal conditions.

## Introduction

The United States is amid a maternal health crisis, with the highest maternal mortality rate among developed nations.^1^ This crisis hits hardest among Indigenous women who have the highest maternal mortality of any race, Black women who face three to four times the mortality risk of white women, and rural women, whose risk is 60% higher than their urban counterparts.^1,2^

Policy efforts have aimed to address these disparities, but few have achieved lasting impact. States face tightening Medicaid budgets, heightened scrutiny around waste, fraud, and abuse, and growing political resistance to expanding benefits and new federal and state policies, including the 2025 One Big Beautiful Bill (OBBB).^1^ In this context, outcome-indexed public-private partnerships (PPPs) offer a strategy for states and managed care organizations (MCO) to safeguard maternal health while linking spending to demonstrated value.

## Navigating maternal equity in a few landscape

New policies and tech developments serve as drivers for these partnerships. The federal interoperability landscape facilitates data exchange and streamlines payments. The ARPA-H “Sprint for Women's Health” and $1B mobilized by the Biden-Harris Administration for women's health research fuel innovation, while venture capital invested $2.6 billion into women's health startups in 2024.^2,3^ This convergence of public and private momentum presents an opportunity to embed equity into innovation. Translating these changes into cost savings and improved outcomes particularly within Medicaid requires deliberate design, evaluation, and accountability.

## FemTech relevance and innovations

The rise of FemTech, technology based solutions for women's health, offers exciting innovations, including virtual doulas, AI-driven risk-assessment tools, and Remote Patient Monitoring (RPM). Self-reported data suggests that these solutions can have a significant impact. A retrospective cohort study of 8989 users found that completing two or more virtual doula appointments was associated with a 20% reduction in cesarean rates and a 68% reduction among Black users.^4^ A fertility-benefits company found that their members had a higher live birth rate of 54.3% vs the national age-adjusted average of 38.4%.^5^ While promising, these company-sponsored studies may have bias and peer-reviewed evidence remains limited. Companies have little to no incentive to share data publicly and may lack the capacity to validate their own data. Independent validation is a necessary prerequisite to widespread adoption.

## The innovation gap: FemTech's Medicaid challenge

Further, these advancements are less accessible to underserved populations. Medicaid, which insures more than 40% of all births in the U.S., receives relatively limited attention from FemTech companies due to its low reimbursement rates and complex state-by-state regulatory structure.^2^ Of the ten highest-funded FemTech companies, only four serve Medicaid patients, highlighting a persistent gap in innovation reach and health equity for low-income populations (Table 1).

**Table 1.** Top 10 funded FemTech companies and insurance coverage offerings.

Company	Total raised (USD M)	Year founded	Accepts insurance	Accepts Medicare/Medicaid
Maven	$412.47	2014	Y	Y
Prelude fertility	$362.50	2016	Y	N
Kindbody	$344.44	2018	Y	N
Advantia health	$216.82	2014	Y	Y
Pomelo care	$170.96	2021	Y	Y
Tia	$157.00	2017	Y	N
Midi health	$150.60	2021	Y	N
Mirvie	$120.30	2018	N	N
Carrot fertility	$116.22	2015	Y	N
Diana health	$101.54	2019	Y	Y

Based on data available on Pitchbook and company websites, accessed January 20, 2026.

FemTech market map determined through a filter of US based FemTech companies with Primary Industry Codes of Clinics/Outpatient Services and Healthcare Services.

Building PPPs, and serving the Medicaid market, is far from simple. One company, designed to serve low-income Black and Brown communities, cited challenges in navigating complex patient needs, restrictive data-sharing environments, and minimal reimbursement—factors that made partnerships unsustainable.^6^

These are not isolated issues. Private-sector incentives continue to favor high-margin markets such as employer-sponsored insurance and self-pay models. Regulatory fragmentation and limited billing pathways complicate implementation and scalability across the Medicaid landscape. Privacy concerns, especially around tools that collect sensitive data, like intimate partner violence, add further risk. Given these profound barriers, sustained public investment is required to incentivize entry for innovators and establish a coordinated pathway for FemTech adoption within the Medicaid system.

## Recommendations and policy actions

Private companies and policymakers can catalyze PPPs in FemTech by aligning tools across the innovation lifecycle—using Advanced Research Projects Agency for Health (ARPA-H) to fund R&D and bridge commercialization gaps, the Transforming Maternal Health (TMaH) model to test solutions in care settings and collect data, and Section 1115 waivers to scale technologies through Medicaid as a base.

ARPA-H provides early-stage funding for high-risk, high-reward research; it’s Sprint for Women's Health program supports advancements, like AI-driven diagnostics. While traditionally confined to early research, ARPA-H's mandate should evolve to include commercialization-stage support, particularly in sectors like FemTech, where market entry is often stymied by long validation cycles and uncertain reimbursement pathways.

To boost private-sector participation in ARPA-H, policymakers could pair it with advanced market commitments—tools that guarantee payment if companies meet specific outcomes. Traditionally used for vaccines, this mechanism offers FemTech firms the market certainty they lack. ARPA-H could facilitate outcomes-based linkages between startups and state Medicaid programs, where service purchase is contingent on program-specific metrics—for example, reducing postpartum hospitalizations through remote pre-eclampsia monitoring. This approach reduces financial uncertainty for innovators while aligning private incentives with clearly defined public health outcomes.

Once early-stage innovations are validated, the TMaH Model serves as a bridge between innovation and scale, enabling real-world testing and coordination within Medicaid. This CMS initiative allows specific states to fund local agencies with the goal of improving maternal health for Medicaid and CHIP enrollees.^2^ TMaH funds can foster PPPs by incentivizing collaboration among state Medicaid agencies, FemTech companies, hospitals, and community organizations.

To bolster the TMaH Model's emphasis on collaboration, policymakers should build a maternal health trust using the nation's growing interoperability ecosystem including Trusted Exchange Framework and Common Agreement (TEFCA) and CMS Interoperability regulations. While TEFCA provides the national governance framework and technical infrastructure for exchange, CMS Interoperability Regulations provide the regulatory mandate necessary to ensure that public payers and participating providers participate in data-sharing.^7^ Critics note that uneven vendor participation and persistent data silos are barriers in practice for these interoperability frameworks. A dedicated maternal health data trust could strengthen incentives. Companies could contribute data in exchange for access to de-identified, aggregate outcome and payment data from partner providers, allowing them to improve their product.^8^ Simultaneously, states could link outcomes to reimbursements and use the data to rigorously evaluate FemTech performance. By collecting both intervention-specific metrics, like preterm birth rate, and equity-focused metrics, like improvements in social determinants of health (SDOH) scores, policymakers can measure the impact of FemTech on maternal health disparities.

Finally, to bring successful pilots to scale, policymakers may consider a Preferred Innovation pathway to strengthen the Medicaid Section 1115 waiver process—one of the few tools that offers states the flexibility to pilot and expand innovative models of care within Medicaid. The current process is slow and fragmented, requiring each state to issue its own request for proposals and vet vendors, often delaying implementation by months or years.^9^

An alternative is to create a Preferred Innovation Vendor designation within CMS, modeled after the FDA's Fast Track. This centralized status would pre-approve FemTech companies that show evidence of impact and commitment to serving Medicaid populations. In return, they would gain access to waiver templates and enhanced reimbursement pathways. For states and MCOs, this would mean access to a vetted pipeline of equity-focused tools, ready for rapid deployment, evaluation, and scale.

## Conclusion

In the current landscape defined by federal budget uncertainty and intensified spending scrutiny maternal health innovation faces substantial barriers. Aligning existing mechanisms such as ARPA-H, the TMaH Model, and Section 1115 waivers is therefore not a call for unchecked expansion, but a strategy for disciplined experimentation: testing which innovations delivers measurable value, for whom, and at what cost. By creating a coordinated pathway, the nation can incentivize innovators while safeguarding maternal health gains.

## Supplementary Material

qxag038_Supplementary_Data
